# Overexpression of *oHIOMT* results in various morphological, anatomical, physiological and molecular changes in switchgrass

**DOI:** 10.3389/fpls.2024.1379756

**Published:** 2024-06-17

**Authors:** Yanhua Huang, Xianzhi Lai, Changfa Liu, Wentao Zhu, Yongren Hao, Zehui Zheng, Kai Guo

**Affiliations:** Biology Institute, Qilu University of Technology (Shandong Academy of Sciences), Jinan, China

**Keywords:** *HIOMT*, melatonin, concentration-dependent, photosynthesis, salt tolerance, lignin content

## Abstract

**Introduction:**

Melatonin (N-acetyl-5-methoxytryptamine) is a molecule implicated in multiple biological functions, but exerts contrasting effects on plants owing to concentration differences. Hydroxyindole O-methyltransferase (HIOMT), which catalyzes the last step of melatonin synthesis, plays a crucial role in this context.

**Methods:**

Transgenic switchgrass overexpressing *oHIOMT* with different melatonin levels displayed distinct morphological changes in a concentration-dependent manner. In this study, we divided the transgenic switchgrass into two groups: melatonin-moderate transgenic (MMT) plants and melatonin-rich transgenic (MRT) plants. To determine the concentration-dependent effect of melatonin on switchgrass growth and stress resistance, we conducted comparative morphological, physiological, omics and molecular analyses between MMT, MRT and wild-type (WT) plants.

**Results:**

We found that *oHIOMT* overexpression, with moderate melatonin levels, was crucial in regulating switchgrass growth through changes in cell size rather than cell number. Moderate levels of melatonin were vital in regulating carbon fixation, stomatal development and chlorophyll metabolism. Regarding salt tolerance, melatonin with moderate levels activated numerous defense (e.g. morphological characteristics, anatomical structure, antioxidant enzymatic properties, non-enzymatic capacity and Na^+^/K^+^ homeostasis). Additionally, moderate levels of *oHIOMT* overexpression were sufficient to increase lignin content and alter monolignol compositions with an increase in the S/G lignin ratio.

**Discussion:**

Taken together, *oHIOMT* overexpression in switchgrass with different melatonin levels resulted in morphological, anatomical, physiological and molecular changes in a concentration-dependent manner, which characterized by stimulation at low doses and inhibition at high doses. Our study presents new ideas and clues for further research on the mechanisms of the concentration-dependent effect of melatonin.

## 1 Introduction

Melatonin (N-acetyl-5-methoxytryptamine) is a tryptophan-derived molecule with pleiotropic activities. It is ubiquitously present in almost all living organisms, including bacteria, fungi, plants and animals (Ma et al., 2022). The biosynthetic pathway of melatonin has been well studied in animals, where tryptophan transforms into melatonin through the catalytic action of four synthesizers: tryptophan decarboxylase (TDC), tryptamine-5-hydroxylase (T5H), arylalkylamine N-acetyltransferase (AANAT) and hydroxyindole O-methyltransferase (HIOMT) (Reiter, 1991). In a wide range of plant species, melatonin synthesis from tryptophan is further catalyzed by six enzymes (TDC, T5H, tryptophan 5-hydroxylase [TPH], aromatic-L-amino-acid decarboxylase [TDC/AADC], serotonin N-acetyltransferase (SNAT), N-acetylserotonin O-methyltransferase [ASMT] or caffeic acid O-methyltransferase [COMT]), which are related to four different routes (Back et al., 2016). In animals as well as in plants, SNAT enzyme catalyzes the conversion of serotonin to N-acetylserotonin. Finally methylation of N-acetylserotonin by the HIOMT also called as ASMT leads to the formation of melatonin (Khan et al., 2020). Thus, HIOMT acts as the rate-limiting enzyme in the melatonin biosynthesis.

Melatonin has been identified in numerous plant species, with its content varying across plant species, organs and environmental conditions, ranging from a few pg/g to over 200 μg/g. Elevated melatonin levels are found in mature seeds (Zhang et al., 2022a). Melatonin has been extensively validated as a regulator of plant growth and development, including seed germination (Chen et al., 2021), seedlings growth (Liu et al., 2022), root development (Yang et al., 2021), flowering and fruit production (Usanmaz et al., 2022). More importantly, melatonin was found to have similar properties to indole-3-acetic acid (IAA). Its effects as growth promoter were dose and tissue-dependent; stimulating plant growth at low concentrations and decreasing it at high doses (Mir et al., 2020). Accumulating evidences from recent studies have indicated that the optimal melatonin concentrations required to regulate plant growth, development and defense differ greatly (Lv et al., 2021; Khan et al., 2022a). It is, therefore, of great significance to explore concentration-dependent effects of melatonin on plants.

In addition to promoting plant growth and developmental, melatonin plays an important role in responses to biotic (Thangaraj et al., 2022; Fathi et al., 2023) and abiotic stresses (Ahmad et al., 2023; Ghorbani et al., 2023; Xu et al., 2023). Extensive studies have revealed the crucial role of melatonin in plant salt tolerance (Ayyaz et al., 2022). Melatonin can promote seed germination and seedling growth (Chen et al., 2021), enhance physiological activity (Bhavithra et al., 2021) and produce increased yield (Khan et al., 2022b) in plants under salinity-induced stress. The potential regulatory mechanisms has been extensively explored at physiological, biochemical and molecular levels. The primary function of melatonin in response to salt stress is that it acts as a broad-spectrum antioxidant and a receptor-independent free radical scavenger (Zhang et al., 2022b; Meftahizadeh et al., 2023). Moreover, melatonin enhances salt stress tolerance in plants by promoting photosynthetic capacity (Fathi et al., 2023), flavonoid enrichment (Song et al., 2022) and modulating the expression of defense-related genes (Elsayed et al., 2021). Additionally, melatonin can also mitigate salinity-induced damage by regulating Na^+^/K^+^ homeostasis (Wei et al., 2021; Zahedi et al., 2021). Therefore, melatonin can trigger a number of defense responses or strategies of plants to salt stress.

In recent years, more functions of melatonin have been identified in higher plants, mainly its roles as a circadian rhythms regulator. In vertebrates, melatonin is rhythmically secreted by the pineal gland and is primarily involved in regulation of circadian rhythm (Li et al., 2023). Melatonin levels in vertebrates were significantly higher during the dark period than during the light period (Moderie et al., 2021). Consistent with these findings, melatonin in plants also exhibits diurnal rhythms, adapting to the daily light–dark cycle, with high production at night (Huang et al., 2017). Moreover, key genes for all enzymes involved in the melatonin synthesis pathway in plants show peak expression at night (Ahn et al., 2021). Recently, melatonin-regulated circadian rhythms were found to be important for plants to respond to various abiotic stresses. For example, melatonin improves the growth of hulless barley (*Hordeum vulgare* L.) under cold stress by influencing the expression rhythms of circadian clock genes (Chang et al., 2021). Additionally, melatonin restores rhythmical redox homeostasis (Chang et al., 2022) and acts as a darkness signal in circadian stomatal closure (Li et al., 2020). The role of melatonin in regulating circadian rhythms is highly significant for improving plant adaptation to diverse environmental stresses.

Previously, we introduced the homologous ovine genes *oAANAT* and *oHIOMT* into switchgrass and obtained transgenic switchgrass with enhanced melatonin production (Huang et al., 2017). The transgenic switchgrass with overexpressed *oHIOMT* (OE-*oHIOMT*) exhibited two distinct phenotypes related to melatonin concentration. However, our previous study did not explore the underlying mechanism. Recent advancements in multi-omics analyses, which integrates various genomic, transcriptomic, proteomic and metabolomic analyses, have been widely accepted as powerful tools for discovering the mechanism of action of melatonin (Huang et al., 2022). In the present study, we conducted RNA sequencing and proteomic analysis (based on isobaric tags for relative and absolute quantitation) to identify differentially expressed genes and proteins in OE-*oHIOMT* transgenic lines with varying melatonin levels. Finally, we detected the concentration-dependent effect of melatonin on switchgrass growth, photosynthetic characteristics, enzyme activities and salt resistance. This study will elucidate the possible molecular mechanisms underlying oHIOMT and concentration-dependent effects of melatonin on plant growth, development and defense, providing an important starting point for further analyses.

## 2 Materials and methods

### 2.1 Plant materials

The transgenic switchgrass with overexpressed *oHIOMT* (OE-*oHIOMT*) were regenerated from embryogenic calli following a modified *Agrobacterium*-mediated transformation protocol (Huang et al., 2017). OE-*oHIOMT* transgenic lines were classified into two groups: melatonin-moderate transgenic (MMT) plants and melatonin-rich transgenic (MRT) plants. Wild-type (WT) plants regenerated from untransformed calli were controls. MMT, MRT and WT plants were grown in a greenhouse under a 16/8 light/dark period, temperature of 28°C ± 3°C day and 18°C ± 2°C night, relative humidity of 65% ± 5% and light intensity of 200 μmol m^-2^s^-1^. Switchgrass development was divided into five elongation (E1, E2, E3, E4 and E5) and three reproductive (R1, R2 and R3) stages, following the criteria outlined by Hardin et al. (2013).

### 2.2 Measurement of growth traits and melatonin

MMT, MRT and WT plants were subjected to phenotypic analysis, comparing and calculating tiller number, plant height, stem diameter, leaf blade length and leaf width. At the E5 stage, fully expanded mature leaves of internode 3 (I3) from the MMT, MRT and WT plants were selected for leaf length and width comparison. I3 was used for stem diameter measurements. When switchgrass reached the R3 stage, leaves of MMT, MRT and WT plants collected simultaneously were used for determining melatonin levels. Melatonin was extracted and detected using high-performance liquid chromatography (HPLC) following our previously reported method (Huang et al., 2017). All measurements were conducted in three biological replicates, and each replicate containing three plants.

### 2.3 Transcriptomic and proteomic analyses

Mature leaves of I3 from MMT, MRT and WT plants were subjected to transcriptomic and proteomic analyses. Samples from three different plants were pooled for each biological replicate, and three biological replicates were performed. Transcriptomic sequencing was performed by Novogene Technology Co., Ltd. (Beijing, China). Briefly, RNA was extracted using the TRIzol method and tested for purity, concentration and integrity. We obtained high-quality RNA suitable for cDNA library construction. The cDNA library was sequenced using an Illumina Hiseq platform, and clean reads were aligned to the switchgrass reference genome sequence (https://phytozome-next.jgi.doe.gov/info/Pvirgatum_v4_1). Data were analyzed following our previously reported methods (Huang et al., 2022). For proteomic analysis, 150 mg of powder from each sample was used as the input material for protein preparation. Isobaric tags for relative and absolute quantification (iTRAQ) labeling of peptides, HPLC fractionation and NanoLC-MS/MS analysis were conducted by Novogene Technology Co., Ltd. (Beijing, China). The raw data were analyzed with ProteinPilot Version 4.2 using the Paragon search engine against the P. virgatum Version 4.1 database. Proteins with a fold change of >1.5 and *p*-value of < 0.05 were considered significantly differentially expressed proteins (DEPs).

### 2.4 Histological and scanning electron microscopy analyses

Mature I3 leaves collected from MMT, MRT and WT plants at Rl stage were used for histological and SEM analyses. For histological analyses, the leaves were fixed in formalin acetoalcohol (FAA) solution for 24 h, dehydrated in ethanol and infiltrated with paraffin, as described previously (Cui et al., 2020). Cross-sections were cut using a microtome and stained with safranin-*O*-fast green to observe cell morphology. Photographs of the sections were taken using an Olympus BX-51 compound microscope, and data were analyzed using Image-Pro Plus Version 6.0 software. For SEM observations, samples were fixed, dehydrated and dried at critical points, following the previously reported method (Pathan et al., 2010). Afterwards, the samples were mounted on a stub, coated with gold and examined under a Hitachi S3400N scanning electron microscope. The area, diameter and number of different cells were calculated using Image-Pro Plus Version 6.0 software. All measurements were conducted in three biological replicates, and each replicate containing four plants.

### 2.5 Salt stress treatments

At the R1 stage, MMT, MRT and WT plants were subjected to a salt-stress test. For the leaf senescence assay, mature I3 leaves were collected, cut into small pieces (~4-cm long), and floated in different concentrations of NaCl solution (0, 200 and 400 mM) for 14 days. The phenotypic changes in leaves were compared and recorded. In the salt-stress test, uniform tillers were selected for sand culture in Hoagland’s nutrient solution supplemented with 0, 200 and 400 mM NaCl. To evaluate the differences in leaf morphology with and without salt stress, mature leaves of MMT, MRT and WT plants were subjected to histological and SEM analyses following the method described above.

### 2.6 Determination of physiological traits

After 30 days of treatment, mature I3 leaves were used to determine chlorophyll and hydrogen peroxide (H_2_O_2_) contents and antioxidant enzyme activity. Chlorophyll content was measured following a modified method (Lichtenthaler and Wellburn, 1983). Chlorophyll a (Chl a), chlorophyll b (Chl b) and total carotenoid contents were calculated using conventional spectroscopic methods. H_2_O_2_ content was measured using a modified method (Brennan and Frenkel, 1977). The enzyme activities of superoxide dismutase (SOD), catalase (CAT) and ascorbate peroxidase (APX) were measured spectrophotometrically as previously described (Nakano and Asada, 1981; Dhindsa et al., 1982), with absorbance values recorded at 560, 240 and 290 nm, respectively. Moreover, the stems of MMT, MRT and WT plants at the R1 stage were sampled for lignin content determination and monomer composition analysis. Lignin content was quantified following the National Renewable Energy Laboratory standard protocols. Lignin monomers were measured following a modified thioacidolysis protocol (Robinson and Mansfield, 2009). Furthermore, switchgrass roots and shoots were harvested for Na^+^ and K^+^ detection. All measurements were conducted in three biological replicates, and each replicate containing three plants.

### 2.7 Quantitative real-time polymerase chain reaction

To confirm differential gene expression in MMT, MRT and WT plants, qRT-PCR assay was conducted. Briefly, RNA from leaves at the E5 stage was extracted using the TRIzol reagent method. Subsequently, the RNA samples were subjected to reverse transcription to obtain cDNA. Primers specific to selected transcripts were designed based on transcriptome sequencing results. The primer sequences are listed in **Supplementary Table 1**. The switchgrass ubiquitin-1 gene (*PvUBQ1*) (FL899020) served as the internal reference gene for the normalization. The qRT-PCR was performed in a 10-mL reaction volume following the manufacturer’s instructions. Three biological experiments with three technical replicates for each sample were conducted.

### 2.8 Statistical analyses

All experiments were independently performed with at least three replications. Data are presented as mean ± standard error (SE). Data from each trait were subjected to analysis of variance using SPSS software (Version 18.0, IBM, Armonk, NY, USA). The differences were compared using Duncan’s test with a significance level of *p* < 0.05 or *p* < 0.01. SEs have been provided in all tables and figures as appropriate.

## 3 Results

### 3.1 *oHIOMT* overexpression in switchgrass results in morphological changes

**Figure 1:**
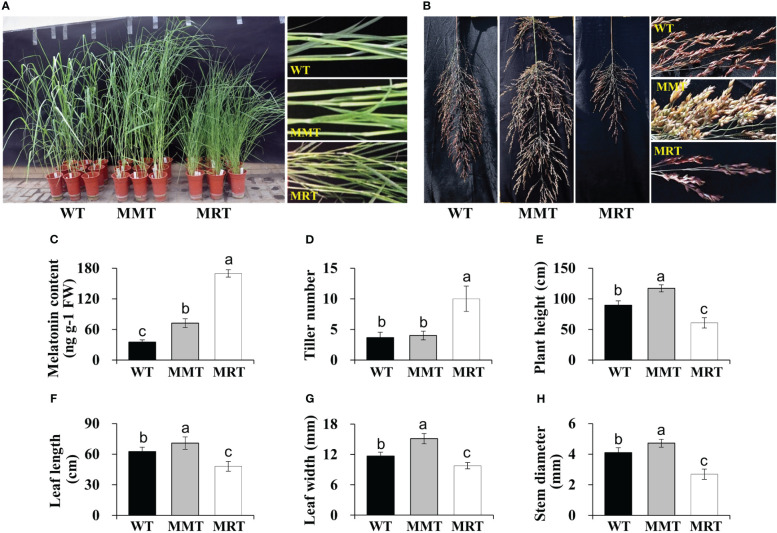
Phenotypic analysis of transgenic switchgrass overexpressing *oHIOMT*. **(A)** Growth performance of transgenic switchgrass. **(B)** Morphological development of transgenic switchgrass spikes. Statistical analyses of **(C)** melatonin, **(D)** tiller number, **(E)** plant height, **(F)** leaf length, **(G)** leaf width and **(H)** stem diameter. WT, wild type; MMT, melatonin-moderate transgenic switchgrass; MRT, melatonin-rich transgenic switchgrass. Values are presented as means ± SE (n = 9). Different letters above the bars indicate significant differences at *p* < 0.01 (Duncan’s test).

OE-*oHIOMT* transgenic switchgrass showed growth- and development-associated phenotypic differences compared with WT plants (**Figures 1A, B**). Based on their melatonin level, OE-*oHIOMT* transgenic lines were classified into two groups: MMT (2.07-fold of WT) and MRT (4.84-fold of WT) (**Figure 1C**). Compared with WT plants, the tiller number of MMT and MRT plants were increased by 9% and 173%, respectively (**Figure 1D**). Interestingly, MRT plants showed a severely stunted phenotype, while MMT plants showed obvious advantages in growth, as compared to the WT plants (**Figures 1D–H**). For example, the plant height and leaf width of MRT plants increased by 31% and 29% than WT plants, respectively; whereas the corresponding indicators of MRT plants were significantly decreased by 32% and 17% than WT plants, respectively (**Figures 1E, G**). Therefore, transgenic switchgrass overexpressing *oHIOMT* with different melatonin levels displayed distinct morphological changes in a concentration-dependent manner.

### 3.2 Transcriptomic and proteomic analyses of transgenic switchgrass

**Figure 2:**
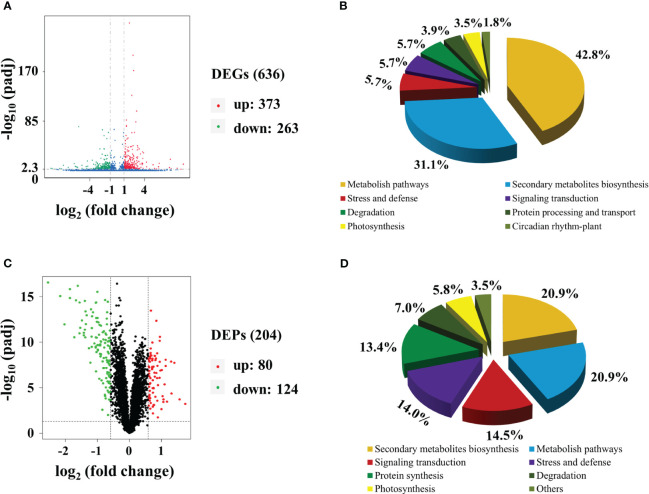
Transcriptomic and proteomic analyses of MMT and MRT switchgrass. Volcano plots of **(A)** DEGs and **(C)** DEPs. Functional classifications of **(B)** DEGs and **(D)** DEPs. MMT, melatonin-moderate transgenic switchgrass; MRT, melatonin-rich transgenic switchgrass; DEG, differentially expressed gene; DEP, differentially expressed protein.

Transcriptomic analysis was conducted to identify changes in gene expression between MMT and MRT plants. We identified 636 differentially expressed genes (DEGs), comprising 373 upregulated and 263 downregulated genes (**Figure 2A**). Gene Ontology (GO) enrichment analysis indicated that the significantly enriched biological process (BP), cellular component (CC) and molecular function (MF) terms were “aromatic compound biosynthetic process”, “catalytic complex” and “ion binding”, respectively (**Supplementary Figure 1A**). The top two significantly enriched Kyoto Encyclopedia of Genes and Genomes (KEGG) pathways were “metabolite pathways” and “secondary metabolite biosynthesis”, accounting for 43% and 31% of the total identified DEGs, respectively (**Figure 2B**). Additionally, environmental adaption pathways such as “stress and defense” (zma00073), “photosynthesis” (zma00195) and “circadian rhythm–plant” (zma04712) were also found. We also conducted proteomic analysis to identify the altered proteins in MMT switchgrass compared with MRT plants. Based on the proteomic analysis, 204 DEPs were obtained, including 80 up-accumulated and 124 down-accumulated proteins (**Figure 2C**). These DEPs were mainly related to “secondary metabolite biosynthesis”, “stress and defense” and “photosynthesis”, as revealed by GO enrichment and KEGG analyses (**Figure 2D**; **Supplementary Figure 1B**).

### 3.3 Concentration-dependent effect of melatonin on cell growth and development

**Figure 3:**
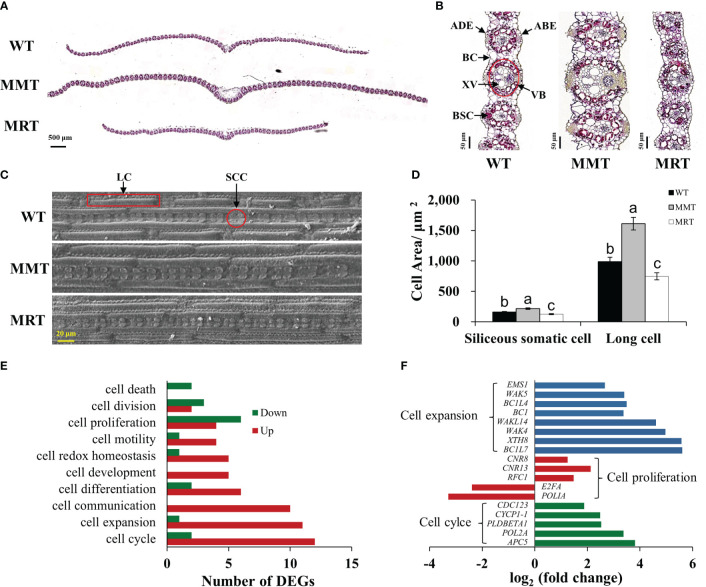
Anatomical and molecular analyzes of MMT and MRT switchgrass cells. **(A)** Cross-sectional anatomical structure (scale bar = 500 μm). **(B)** Kranz structure from cross sections (scale bar = 50 μm). **(C)** Morphological characteristics and **(D)** statistical analyses of SCCs and LCs (scale bar = 20 μm). **(E)** Classifications of DEGs involved in cell growth and development. **(F)** Expression level analysis of genes related to cell expansion, cell proliferation and cell cycle. WT, wild type; MMT, melatonin-moderate transgenic switchgrass; MRT, melatonin-rich transgenic switchgrass; DEG, differentially expressed gene; LC, long cell; SCC, siliceous somatic cell. Values are presented as means ± SE (n = 12). Different letters above the bars indicate significant differences at *p* < 0.01 (Duncan’s test).

OE-*oHIOMT* switchgrass with different melatonin levels showed phenotypic changes in a concentration-dependent manner. To determine the cellular basis of this phenotype, histological and SEM analyses were conducted. The sizes of abaxial epidermal (ABE) cells, adaxial epidermal (ADE) cells, bundle sheath cells (BSCs), bulliform cells (BCs), vascular bundles (VBs), xylem vessels (XVs), siliceous somatic cells (SSCs) and long cells (LCs), were significantly increased in the MMT plants compared with control plants. Conversely, the sizes of BSCs, ADE cells, and BCs of MRT plants were significantly decreased by 77%, 54% and 37% than WT plants, respectively (**Figures 3A–D** and **Supplementary Table 2**). However, no significant difference was observed in the number of BSCs per bundle and VBs per cross-section between MMT, MRT and WT plants.

Based on transcriptomic data, we identified 10 enriched GO terms related to cell growth and development in MMT plants. Among these, “cell cycle”, “cell expansion” and “cell communication” were the most abundant. Notably, all DEGs in the “cell communication” and “cell development” groups were upregulated, whereas the DEGs in the “cell death” group were downregulated. Moreover, most DEGs involved in “cell expansion” and “cell cycle” were upregulated. Conversely, downregulated DEGs significantly surpassed upregulated DEGs in the “cell proliferation” and “cell division” groups (**Figure 3E**). Among these DEGs, seven cell expansion–related genes and two cell cycle–related genes exhibited increased expression levels by over 3.00-fold. The expressions of three genes (cell number regulator [*CNR8*, *CNR13*] and replication factor C subunit 1 [*RFC1*]) involved in cell proliferation were activated, whereas two genes (DNA polymerase I A [*POLIA*] and transcription factor E2FA [*E2FA*]) were repressed in MMT plants (**Figure 3F**). These results suggested that *oHIOMT* overexpression with moderate melatonin levels regulates switchgrass growth through changes in cell size rather than cell number.

### 3.4 Concentration-dependent effect of melatonin on photosynthesis

**Figure 4:**
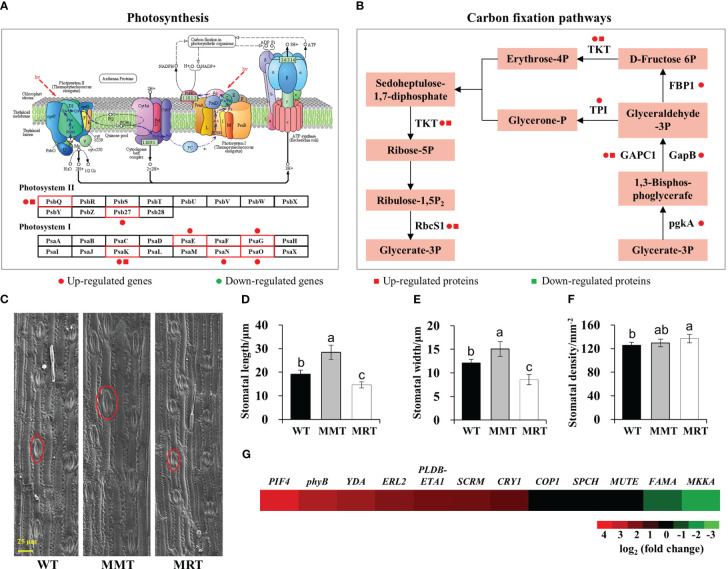
Photosynthesis analysis of MMT and MRT switchgrass. **(A)** DEGs and DEPs involved in photosynthesis and **(B)** carbon fixation pathways. **(C)** Stomatal morphological characteristics, **(D)** length, **(E)** width and **(F)** density analysis of MMT, MRT and WT plants (scale bar = 25 μm). **(G)** Expression profiles of genes involved in stomatal development in MMT plants compared with MRT plants. WT, wild type; MMT, melatonin-moderate transgenic switchgrass; MRT, melatonin-rich transgenic switchgrass. Values are presented as means ± SE (n = 12). Different letters above the bars indicate significant differences at *p* < 0.01 (Duncan’s test).

To further elucidate the concentration-dependent effects of melatonin on plant growth and development, we performed integrated transcriptomic and proteomic analyses. Compared with MRT plants, MMT plants exhibited a significant upregulation of some DEGs and DEPs enriched in photosynthesis and carbon fixation pathways (**Figures 4A, B**). Moreover, MMT switchgrass exhibited distinct advantages in stomatal development (**Figure 4C**). Compared with WT plants, the stomatal length and width of MMT plants were increased by 49% and 25%, respectively; whereas the corresponding indicators of MRT plants were significantly decreased by 24% and 30%, respectively (**Figures 4D, E**). However, MMT plants showed no significant differences in stomatal density, MRT plants exhibited a significant increase, as compared to the WT plants (**Figure 4F**). Based on transcriptomics data, we identified nine DEGs related to stomatal development in MMT plants compared to MRT plants (**Figure 4G**). Among the seven upregulated genes, phytochrome-interacting factor 4 (*PIF4*) gene and phytochrome B (*phyB*) genes maintained high expression levels in MMT plants. These results indicated that *oHIOMT* overexpression with moderate melatonin levels enhances photosynthetic capacity by activating the carbon fixation pathway and promoting stomatal development.

### 3.5 Concentration-dependent effect of melatonin on salt tolerance of switchgrass

**Figure 5:**
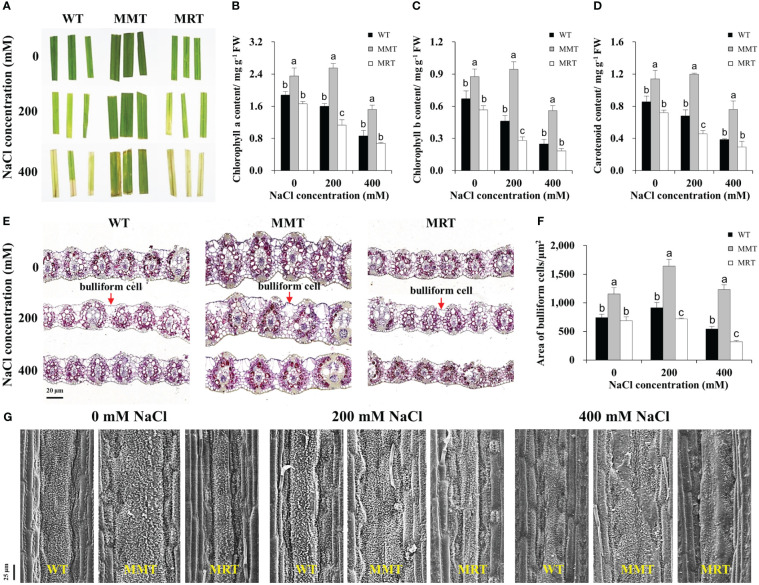
Responses of MMT, MRT and WT switchgrass to various concentrations of NaCl treatment. **(A)** Leaf senescence assay of MMT, MRT and WT plants. **(B)** Chlorophyll a, **(C)** chlorophyll b and **(D)** total carotenoid contents. **(E)** Cross-sectional anatomical structure of MMT, MRT and WT plants under 0, 200 and 400 mM NaCl treatment (scale bar = 20 μm). **(F)** Area of bulliform cells. **(G)** Analysis of waxy structure adaxial on the adaxial surface (scale bar = 25 μm). WT, wild type; MMT, melatonin-moderate transgenic switchgrass; MRT, melatonin-rich transgenic switchgrass. Values are presented as means ± SE (n = 3). Different letters above the bars indicate significant differences at *p* < 0.01 (Duncan’s test).

We compared the salt tolerance of MMT, MRT and WT plants via leaf senescence assay. As shown in **Figure 5A**, the damage caused by salt stress was visualized by the degree of bleaching in leaf tissues. Leaves from MMT plants showed better salt tolerance by retaining green color even at 400 mM NaCl, whereas those from MRT and WT plants exhibited severe chlorosis and bleaching. Moreover, we measured Chl a, Chl b and total carotenoid contents of MMT, MRT and WT plants under different salt stress conditions. Compared with WT plants, the Chl a, Chl b and total carotenoid contents in MMT plants were significantly increased, whereas the corresponding contents in MRT plants were decreased. Notably, low salinity (200 mM NaCl) induced an increase in Chl a and Chl b contents in MMT plants (**Figures 5B–D**).

To further elucidate the concentration-dependent effects of melatonin on plant salt tolerance at the cellular level, we conducted histological and SEM analyses. BCs active in the folding and unfolding of leaves, store more water for plant usage under salt stress (Matschi et al., 2020). Significant morphological differences were observed in the BCs of MMT, MRT and WT plants under salt stress (**Figure 5E**). Compared with WT plants, the areas of BCs were increased by 55% and 126% in MMT plants under 0 and 400 mM NaCl conditions, respectively, whereas the corresponding levels in MRT plants were decreased by 8% and 41%, respectively (**Figure 5F**). In addition to BCs, plant surface waxes can protect the plants against environmental stresses. As shown in **Figure 5G**, the leaf surfaces of MMT plants were more densely covered with lamellar wax crystals with or without salt stress compared with those of WT plants. Conversely, fewer and shorter wax crystals were observed in the leaf surfaces of MRT plants. Further analysis was conducted to evaluate the expression of wax synthesis–related genes. We identified eleven DEGs, encompassing eight genes involved in very long chain fatty acid (VLCFA) synthesis, two genes related to alkane synthesis and one gene associated with primary alcohol synthesis. Additionally, we selected five genes for qRT–PCR analysis. As shown in **Supplementary Figure 2**, the qRT–PCR results correlated well with the transcriptomic results. The expressions of two 3-Ketoacyl-CoA synthase genes (*KCS6* and *KCS19*) of MMT plants were highly upregulated under salt stress, as compared to the WT and MRT plants. These results indicated that *oHIOMT* overexpression with moderate melatonin levels enhances salt tolerance by regulating BCs and wax formation.

### 3.6 Concentration-dependent effect of melatonin on the antioxidant defense system

**Figure 6:**
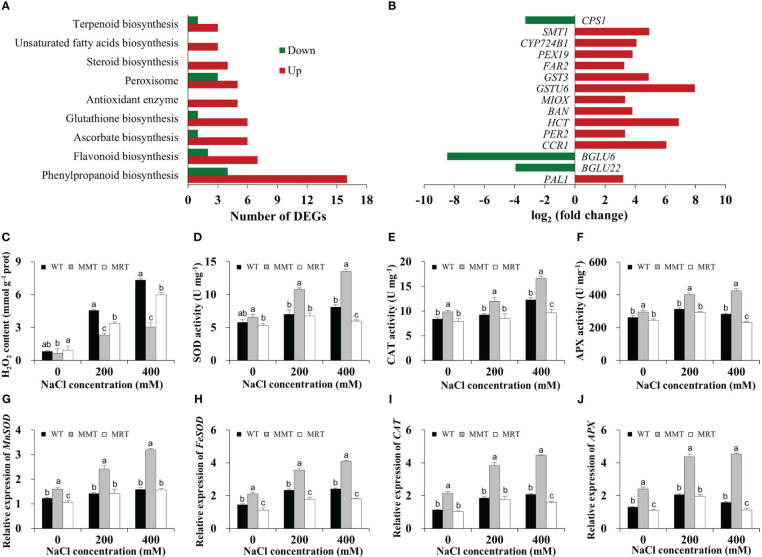
Antioxidant defense system analysis of MMT, MRT and WT switchgrass under salt stress conditions. **(A)** Classifications of DEGs involved in the antioxidant defense system. **(B)** Expression level analysis of genes involved in non-enzymatic antioxidant systems. Statistical analyses of **(C)** H_2_O_2_ content, **(D)** SOD activity, **(E)** CAT activity and **(F)** APX activity. Expression levels of **(G)** *MnSOD*, **(H)** *FeSOD*, **(I)** *CAT* and **(J)** *APX*. WT, wild type; MMT, melatonin-moderate transgenic switchgrass; MRT, melatonin-rich transgenic switchgrass; DEG, differentially expressed gene. Values are expressed as means ± SE (n = 3). Different letters above the bars indicate significant differences at *p* < 0.01 (Duncan’s test).

The transcriptomic results revealed through GO and KEGG enrichment analyses showed that many DEGs were markedly enriched in enzymatic and non-enzymatic antioxidant systems. Sixteen DEGs were categorized under the phenylpropanoid biosynthesis pathway in MMT plants (**Figure 6A**). Moreover, some genes related to flavonoid, glutathione, steroid, ascorbate and terpenoid biosynthesis were significantly upregulated in the MMT plants compared with MRT plants. For example, glutathione S-transferase U6 (*GSTU6*) functioning in glutathione biosynthesis and shikimate O-hydroxycinnamoyltransferase (*HCT*) involved in flavonoid biosynthesis increased expression levels by more than 6.80-fold (**Figure 6B**).

Next, we assayed the H_2_O_2_ content in MMT, MRT and WT plants with or without salt stress. Compared with WT plants under normal conditions, the H_2_O_2_ content of the WT plants under 400 mM NaCl conditions were increased by 8.89-fold, whereas that of MMT plants increased by only 3.69-fold (**Figure 6C**). Moreover, the activities of SOD, CAT and APX of MMT plants increased significantly and were significantly higher than that of the MRT and WT plants under salt stress conditions (**Figures 6D–F**). Consistently, four anti-oxidant enzyme genes (Mn-superoxide dismutase [*MnSOD*], Fe-superoxide dismutase [*FeSOD*], *CAT* and *APX*) were significantly upregulated in MMT plants compared with WT plants, with an approximately 1.88-fold increase in expression (**Figures 6G–J**). These results revealed that *oHIOMT* overexpression with moderate melatonin levels can regulate both enzymatic and non-enzymatic antioxidant systems to reduce oxidative damage.

### 3.7 Concentration-dependent effect of melatonin on Na^+^ and K^+^ accumulation

**Figure 7:**
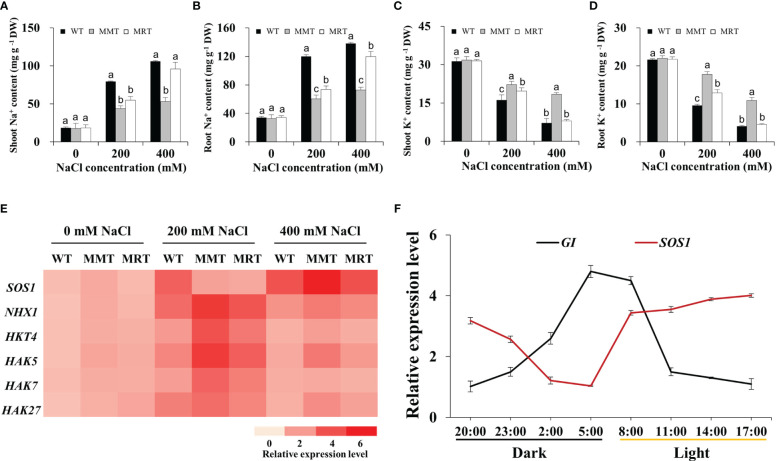
Effects of melatonin on Na^+^ and K^+^ accumulation. **(A)** Na^+^ content in shoots. **(B)** Na^+^ content in roots. **(C)** K^+^ content in shoots. **(D)** K^+^ content in roots. **(E)** Expression level analysis of two Na^+^/H^+^ type antiporter genes (*SOS1* and *NHX1*) and four potassium transporter genes (*HKT4*, *HAK5*, *HAK7* and *HAK27*) under salt stress. **(F)** The expression of the Na^+^/H^+^-type antiporter gene *SOS1* and the circadian clock output gene *GI* changes during a 24-h period. WT, wild type; MMT, melatonin-moderate transgenic switchgrass; MRT, melatonin-rich transgenic switchgrass. Values are expressed as means ± SE (n = 3). Different letters above the bars indicate significant differences at *p* < 0.01 (Duncan’s test).

To examine whether melatonin enhances salt tolerance in plants by improving Na^+^/K^+^ homeostasis, we measured the Na^+^ and K^+^ contents in the shoots and roots of MMT, MRT and WT plants. These plants showed approximately equal Na^+^ and K^+^ contents under normal conditions but increased Na^+^ concentration and significantly decreased K^+^ concentration under salt stress (**Figures 7A–D**). Na^+^ was mainly accumulated in the roots, whereas K^+^ was mostly accumulated in the shoots. However, MMT switchgrass accumulated significantly higher K^+^ and lower Na^+^ content in all tissues compared with MRT and WT plants. Next, we investigated the expression levels of two Na^+^/H^+^-type antiporter genes (Salt Overly Sensitive 1 [*SOS1*] and Na^+^/H^+^ antiporter 1 [*NHX1*) and four potassium transporter genes (high affinity K^+^ transporter 4 [*HKT4*] and potassium transporter [*HAK5*, *HAK7*, *HAK27*]) with or without salt stress. Notably, the expression level of five genes (*NHX1*, *HKT4*, *HAK5*, *HAK7* and *HAK27*) exhibited an increase followed by a decrease with the increase of NaCl concentration, with a low salinity level (200 mM NaCl) showing the maximum value in all plants (**Figure 7E**). However, *SOS1* gene in MMT and MRT plants exhibited different expression patterns, which were strongly induced by a high salinity level (400 mM NaCl). Notably, *SOS1* has been found to regulate the salt compensation of circadian rhythms by stabilizing GIGANTEA (GI) (Cha et al., 2022).

In this study, four DEGs (*GI*, Late elongated hypocotyl [*LHY*], Circadian clock-associated 1 [*CCA1*] and EARLY FLOWERING 3 [*ELF3*]) involved in the circadian rhythm–plant pathway were significantly altered in MMT plants. Among these DEGs, *GI* transcript levels substantially increased in MMT plants and remained relatively stable at higher salt concentrations (400 mM NaCl) (**Supplementary Figure 3**). We focused on the circadian rhythms of *SOS1* and *GI* expression. As shown in **Figure 7F**, *GI* expression exhibited a diurnal rhythmic profile, with the highest level appearing at 05:00. *SOS1* transcript levels followed the opposite trend, exhibiting a marked decline after 17:00. These results indicated that *oHIOMT* overexpression with moderate melatonin levels improves the salt tolerance of switchgrass by regulating Na^+^/K^+^ homeostasis under salt stress. Notably, SOS1 and GI participated in this regulation, which requires further verification.

### 3.8 Concentration-dependent effect of melatonin on lignin synthesis

**Figure 8:**
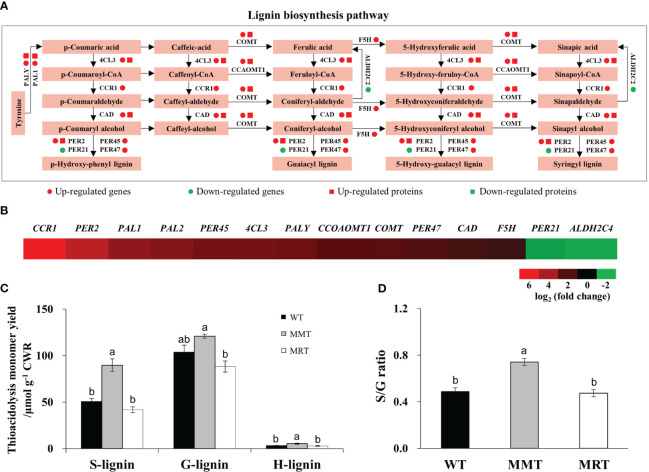
Effects of melatonin on lignin synthesis. **(A)** DEGs and DEPs involved in the lignin biosynthesis pathway. **(B)** Expression profiles of genes involved in lignin biosynthesis in MMT plants compared with MRT plants. **(C)** Lignin content and monolignol compositions. **(D)** S/G ratio. WT, wild type; MMT, melatonin-moderate transgenic switchgrass; MRT, melatonin-rich transgenic switchgrass; DEG, differentially expressed gene; DEP, differentially expressed protein.

Based on integrated transcriptomics and proteomics data, we identified eleven genes and six proteins involved in the lignin biosynthesis pathway of MMT plants (**Figure 8A**). The expression levels of six genes (phenylalanine ammonia-lyase 1 [*PAL1*], 4-coumarate:CoA ligase 3 [*4CL3*], Caffeic acid 3-O-methyltransferase [*COMT*], Caffeoyl coenzyme A 3-O-methyltransferase 1 [*CCoAOMT1*] and Peroxidase 2 [*PER2*]) and their corresponding proteins were significantly upregulated in MMT plants. Four genes (Cinnamoyl-CoA reductase 1 [*CCR1*], Ferulate 5-hydroxylase [*F5H*] and Peroxidase [*PER45*, *PER47*]) in MMT plants were upregulated at the mRNA level without a change in protein abundance. Notably, *CCR1* and *PER2* increased expression levels by more than 4.00-fold in MMT plants, as compared to the WT plants (**Figure 8B**).

Further analysis was conducted to determine the lignin content and monolignol composition of MMT, MRT and WT plants. Compared with WT plants, MMT plants exhibited similar (no significant difference) guaiacyl (G) content but a 78% increase in syringyl (S) and 67% increase in hydroxyphenyl (H) contents (**Figure 8C**). However, there was no significant difference in lignin contents between MRT and WT plants. Moreover, MMT switchgrass exhibited altered monolignol compositions with an increase in the S/G lignin ratio (**Figure 8D**). These results indicated that *oHIOMT* overexpression with moderate melatonin levels was sufficient to increase lignin content and alter monolignol compositions.

## 4 Discussion

Melatonin, a low molecular weight amphiphilic indoleamine signaling molecule, has been identified in numerous plants at distinct levels. Melatonin’s effects on plants vary depending on its concentration. Notably, high and low melatonin concentrations exert contrasting effects on plant morphology and growth (Mir et al., 2020; Khan et al., 2022a). Consistent with these results, we found that transgenic switchgrass overexpressing *oHIOMT* exhibited distinct morphological, anatomical, physiological and molecular changes in a concentration-dependent manner. Exploring the mechanisms behind the concentration-dependent effects of melatonin on plant growth and development is essential. Due to similarity in the chemical structure of melatonin and IAA, it was suggested in initial studies that melatonin has the growth promoting activity probably by increasing auxin levels or showing an auxin-like activity (Arnao and Hernández-Ruiz, 2018). Similarly, melatonin treatment can enhance the accumulation of IAA in *Arabidopsis* (Lv et al., 2021), trifoliate orange (*Poncirus trifoliata* L.) (Hu et al., 2022), soybean (*Glycine max* L.) (Wei et al., 2022). However, some studies demonstrated that melatonin had no effect or negative effect on IAA (Wang et al., 2016; Zia et al., 2019). Accumulating evidences have indicated that melatonin promotes IAA content in a dosage manner. At a certain low concentration, melatonin promotes IAA synthesis, whereas at high levels it reduces IAA production (Wang et al., 2022; Altaf et al., 2023). Apart from the differences in melatonin concentration, it is also important to note that differences exist with regards to specificity of plant species (monocots or dicots) (Kołodziejczyk et al., 2015; Yang et al., 2023) and melatonin treatment (exogenous treatment or transgenic method) (Wei et al., 2022; Zhang et al., 2022c). Therefore, the interaction between melatonin and IAA is complex and has not been fully explored. Further studies elucidating the crosstalk of melatonin with the hormones IAA may shed new light on the mechanism of melatonin-regulated growth in plants.

In addition to regulating plant growth, melatonin also play an important role in regulating photosynthesis in a concentration-dependent manner. For example, the photosynthesis of *Brassica juncea* was significantly improved by exogenous melatonin at 40 μM concentrations (Mir et al., 2020). Likewise, the photosynthetic capacity and related gene expression in tea plant (*Camellia sinensis* L.) were effectively enhanced by exogenous melatonin at 0.2 mM concentrations (Yang et al., 2022). Consistent with these results, we found that melatonin-moderate transgenic (MMT) switchgrass exhibited high photosynthetic activity under normal and salt stress conditions, accompanied by an increase in stomata area and decrease in stomata density. This result has been confirmed in many plant species, such as tomato (*Solanum lycopersicum* L.) (Khan et al., 2023), *Zanthoxylum armatum* (Su et al., 2023) and trifoliate orange (Hu et al., 2022), where the photosynthetic capacity were significantly improved by exogenous melatonin via promoting stomatal area rather than stomatal numbers. Stomata are microscopic pores on the epidermal surface of plants that regulate the exchange of gas (CO_2_ and O_2_) and water vapor with the environment, and play important roles in the interaction between plants and environment (Chen, 2023). Stomatal development and patterning are regulated by both internal genetic programs and environmental cues (Wei et al., 2020). In this study, melatonin improved stomatal traits by regulating the transcription of stomatal development-related genes (e.g. *SCRM*, *SPCH*, *MUTE* and *FAMA*). This result was supported by previous studies (Khan et al., 2019; Huang et al., 2022). In addition, the transcript abundances of most key genes involved in carbon fixation pathways were also changed in OE-*oHIOMT* switchgrass. Similarly, exogenous melatonin treatment also leads to higher expression of genes involved in carbon fixation in kiwifruit (*Actinidia chinensis*) (Liang et al., 2019). Switchgrass is a C4 perennial grass, using an NADP-dependent malic enzyme (NADP-ME) subtype C4 photosynthetic system to fix carbon (Cui et al., 2020). Accumulating evidence has indicated that C4-photosynthetic carbon cycle is an elaborated addition to the C3-photosynthetic pathway, which improves photosynthetic energy conversion efficiency (Baird et al., 2024). Taken together, these results indicate that optimal concentrations of melatonin can enhance photosynthetic capacity by regulating leaf anatomical structures, activating the carbon fixation pathway and promoting stomatal development.

The pragmatic role of melatonin in the regulation of plants circadian rhythms has received much attention in recent times (Huang et al., 2022; Khan et al., 2022a). In plants, the circadian regulation control over daily energy expenditure and enhance plant growth and development even under stress conditions (Steed et al., 2021; Wang et al., 2021; Dakhiya and Green, 2023). Emerging evidences from recent studies have indicated that melatonin enhances plant tolerance to abiotic stress by maintaining the circadian rhythm under adverse conditions (Ahn et al., 2021; Chang et al., 2022). In the present study, the circadian rhythm–plant pathway was significantly enriched in melatonin-moderate transgenic switchgrass. Moreover, melatonin plays a vital role in regulating switchgrass growth, development and salt stress responses via circadian rhythm pathway. Exploring the mechanisms of melatonin–mediated circadian regulation on salt stress responses is essential. The circadian clock output gene *GI*, a switch in photoperiodicity and circadian clock control, regulates salt stress response in plants through the salt overly sensitive (SOS) pathway. Under normal conditions, GI forms a complex with SOS2 to prevent the SOS2-based activation of the SOS1 Na^+^/H^+^ exchanger. Under salt stress, the degradation of the GI protein releases SOS2, which activates the antiporter activity of SOS1 to export Na^+^ and thus confers salt tolerance (Kim et al., 2013). Moreover, SOS1 functions as a salt-specific circadian clock regulator and stabilizes the GI protein to maintain a proper circadian clock period under salinity conditions (Cha et al., 2022). The relationship between the GI and SOS pathways is associated with salt tolerance and circadian clock functions. However, the regulatory effect of SOS1 on GI stabilization has limitations. Under low salt stress, SOS1 regulates salt compensation of circadian rhythms by stabilizing GI protein, whereas high salt-induced degradation of GI disrupts circadian rhythms (Cha et al., 2022). Therefore, achieving an optimal balance between stress responses and circadian rhythm is crucial for salt tolerance in plants. Interestingly, MMT switchgrass showed a relatively stable expression of *GI* and *SOS1* at higher salt concentrations and accumulated significantly higher K^+^ and lower Na^+^ content. SOS1 has been proven to be essential for maintaining the circadian clock under saline conditions by directly protecting GI. As salt stress–induced secondary signaling molecules, ROS are involved in the SOS1-mediated regulation of GI abundance (Cha et al., 2022). Melatonin, acting as a potent free radical scavenger, was essential for SOS1 function to maintain the circadian clock and acquire salt tolerance. We speculated that GI is an important factor linking melatonin-mediated salt stress and the SOS pathway. Melatonin regulates GI stabilization through ROS clearance, while GI regulates circadian rhythms through the posttranslational regulatory mechanisms of the circadian clock. A higher-level regulatory mechanism might be identified by associating pathways previously considered unrelated.

In this study, we demonstrated that overexpression of *oHIOMT* could significantly increase the content of endogenous melatonin in switchgrass. The endogenous melatonin’s effects on switchgrass growth, photosynthetic characteristics, enzyme activities and salt resistance in a concentration-dependent manner. Our study presents a comparative analysis of concentration-dependent effects of melatonin on plant growth, development and stress responses, providing a foundation for further understanding the molecular mechanisms underlying the effects of melatonin in plants.

## Data availability statement

The datasets presented in this study can be found in online repositories. The names of the repository/repositories and accession number(s) can be found in the article/**Supplementary Material**.

## Author contributions

YHH: Writing – review & editing, Writing – original draft. CL: Writing – original draft, Investigation. XL: Writing – original draft, Data curation. WZ: Writing – original draft, Validation. YRH: Writing – original draft, Project administration. KG: Writing – review & editing, Supervision. ZZ: Writing – review & editing.

## References

[B1] Ahmad R. Alsahli A. A. Alansi S. Altaf M. A. (2023). Exogenous melatonin confers drought stress by promoting plant growth, photosynthetic efficiency and antioxidant defense system of pea (*Pisum sativum* L.). Sci. Hortic. 322, 112431. doi: 10.1016/j.scienta.2023.112431

[B2] Ahn H. R. Kim Y. J. Lim Y. J. Duan S. Eom S. H. Jung K. H. (2021). Key genes in the melatonin biosynthesis pathway with circadian rhythm are associated with various abiotic stresses. Plants 10, 129–144. doi: 10.3390/plants10010129 33435489 PMC7827461

[B3] Altaf M. A. Sharma N. Singh J. Samota M. K. Sankhyan P. Singh B.. (2023). Mechanistic insights on melatonin-mediated plant growth regulation and hormonal cross-talk process in solanaceous vegetables. Sci. Hortic. 308, 111570. doi: 10.1016/j.scienta.2022.111570

[B4] Arnao M. B. Hernández-Ruiz J. (2018). Melatonin and its relationship to plant hormones. Ann. Bot. 121, 195–207. doi: 10.1093/aob/mcx114 29069281 PMC5808790

[B5] Ayyaz A. Shahzadi A. K. Fatima S. Yasin G. Zafar Z. U. Athar H.. (2022). Uncovering the role of melatonin in plant stress tolerance. Theor. Exp. Plant Physiol. 34, 335–346. doi: 10.1007/s40626-022-00255-z

[B6] Back K. Tan D. X. Reiter R. J. (2016). Melatonin biosynthesis in plants: multiple pathways catalyze tryptophan to melatonin in the cytoplasm or chloroplasts. J. Pineal. Res. 61 4), 426–437. doi: 10.1111/jpi.12364 27600803

[B7] Baird A. S. Taylor S. H. Reddi S. Pasquet-Kok J. Vuong C. Zhang Y.. (2024). Allometries of cell and tissue anatomy and photosynthetic rate across leaves of C3 and C4 grasses. Plant Cell Environ. 47, 156–173. doi: 10.1111/pce.14741 37876323

[B8] Bhavithra S. M. Kalarani M. K. Senthil A. Kavitha P. S. (2021). Exogenous melatonin on physiological and yield traits of cassava (*Manihot esculenta Crantz*) under salt stress. Madras Agric. J. 108, 1–8. doi: 10.29321/MAJ.10.000472

[B9] Brennan T. Frenkel C. (1977). Involvement of hydrogen peroxide in the regulation of senescence in pear. Plant Physiol. 59, 411–416. doi: 10.1104/pp.59.3.411 16659863 PMC542414

[B10] Cha J. Kim J. Jeong S. Y. Shin G. Ji M. G. Hwang J.. (2022). The Na^+^/H^+^ antiporter SALT OVERLY SENSITIVE 1 regulates salt compensation of circadian rhythms by stabilizing GIGANTEA in *Arabidopsis* . PNAS 119, e2207275119. doi: 10.1073/pnas.2207275119 35939685 PMC9388102

[B11] Chang T. Xi Q. Wei X. Xu L. Wang Q. Fu J.. (2022). Rhythmical redox homeostasis can be restored by exogenous melatonin in hulless barley (*Hordeum vulgare* L. var. nudum) under cold stress. Environ. Exp. Bot. 194, 104756. doi: 10.1016/j.envexpbot.2021.104756

[B12] Chang T. Zhao Y. He H. Xi Q. Fu J. Zhao Y. (2021). Exogenous melatonin improves growth in hulless barley seedlings under cold stress by influencing the expression rhythms of circadian clock genes. Peer J. 9, e10740. doi: 10.7717/peerj.10740 33552735 PMC7831369

[B13] Chen L. (2023). Emerging roles of protein phosphorylation in regulation of stomatal development. J. Plant Physiol. 280, 153882. doi: 10.1016/j.jplph.2022.153882 36493667

[B14] Chen L. Lu B. Liu L. Duan W. Jiang D. Li J.. (2021). Melatonin promotes seed germination under salt stress by regulating ABA and GA3 in cotton (*Gossypium hirsutum* L.). Plant Physiol. Biochem. 162, 506–516. doi: 10.1016/j.plaphy.2021.03.029 33773227

[B15] Cui X. Cen H. Guan C. Tian D. Liu H. Zhang Y. (2020). Photosynthesis capacity diversified by leaf structural and physiological regulation between upland and lowland switchgrass in different growth stages. Funct. Plant Biol. 47, 38–49. doi: 10.1071/FP19086 31578165

[B16] Dakhiya Y. Green R. (2023). The importance of the circadian system for adaptation to heat wave stress in wild barley (*Hordeum spontaneum*). Environ. Exp. Bot. 206, 105152. doi: 10.1016/j.envexpbot.2022.105152

[B17] Dhindsa R. S. Plumb-Dhindsa P. L. Reid D. M. (1982). Leaf senescence and lipid peroxidation: Effects of some phytohormones, and scavengers of free radicals and singlet oxygen. Physiol. Plant 56, 453–457. doi: 10.1111/j.1399-3054.1982.tb04539.x

[B18] Elsayed A. I. Rafudeen M. S. Gomaa A. M. Hasanuzzaman M. (2021). Exogenous melatonin enhances the ROS metabolism, antioxidant defense-related gene expression and photosynthetic capacity of *Phaseolus vulgaris* L. to confer salt stress tolerance. Physiol. Plant 173, 1369–1381. doi: 10.1111/ppl.13372 33619766

[B19] Fathi N. Kazemeini S. A. Alinia M. Mastinu A. (2023). The effect of seed priming with melatonin on improving the tolerance of *Zea mays* L. var saccharata to paraquat-induced oxidative stress through photosynthetic systems and enzymatic antioxidant activities. Physiol. Mol. Plant Pathol. 124, 101967. doi: 10.1016/j.pmpp.2023.101967

[B20] Ghorbani A. Emamverdian A. Pishkar L. Chashmi K. A. Salavati J. Zargar M.. (2023). Melatonin-mediated nitric oxide signaling enhances adaptation of tomato plants to aluminum stress. S. Afr. J. Bot. 162, 443–450. doi: 10.1016/j.sajb.2023.09.031

[B21] Hardin C. F. Fu C. Hisano H. Xiao X. Shen H. Stewart C. N.. (2013). Standardization of switchgrass sample collection for cell wall and biomass trait analysis. Bioenergy Res. 6, 755–762. doi: 10.1007/s12155-012-9292-1

[B22] Hu C. Zheng Y. Tong C. Zhang D. (2022). Effects of exogenous melatonin on plant growth, root hormones and photosynthetic characteristics of trifoliate orange subjected to salt stress. Plant Growth Regul. 97, 551–558. doi: 10.1007/s10725-022-00814-z

[B23] Huang Y. Liu S. Yuan S. Guan C. Tian D. Cui X.. (2017). Overexpression of ovine *AANAT* and *HIOMT* genes in switchgrass leads to improved growth performance and salt-tolerance. Sci. Rep. 7, 12212. doi: 10.1038/s41598-017-12566-2 28939842 PMC5610178

[B24] Huang Y. Zheng Z. Bi X. Guo K. Liu S. Huo X. X.. (2022). Integrated morphological, physiological and omics analyses reveal the arylalkylamine N-acetyltransferase (*AANAT*) gene contributing to growth, flowering and defence in switchgrass (*Panicum virgatum* L.). Plant Sci. 316, 111165. doi: 10.1016/j.plantsci.2021.111165 35151442

[B25] Khan M. Ali S. Manghwar H. Saqib S. Ullah F. Ayaz A.. (2022a). Melatonin function and crosstalk with other phytohormones under normal and stressful conditions. Genes. 13, 1699. doi: 10.3390/genes13101699 36292584 PMC9602040

[B26] Khan T. A. Fariduddin Q. Nazir F. Saleem M. (2020). Melatonin in business with abiotic stresses in plants. Physiol. Mol. Biol. Plants. 26, 1931–1944. doi: 10.1007/s12298-020-00878-z 33088040 PMC7548266

[B27] Khan T. A. Saleem M. Fariduddin Q. (2022b). Melatonin influences stomatal behavior, root morphology, cell viability, photosynthetic responses, fruit yield, and fruit quality of tomato plants exposed to salt stress. J. Plant Growth Regul. 42, 2408–2432. doi: 10.1007/s00344-022-10713-2

[B28] Khan T. A. Saleem M. Fariduddin Q. (2023). Melatonin influences stomatal behavior, root morphology, cell viability, photosynthetic responses, fruit yield, and fruit quality of tomato plants exposed to salt stress. J. Plant Growth Regul. 42, 2408–2432. doi: 10.1007/s00344-022-10713-2

[B29] Khan M. N. Zhang J. Luo T. Liu J. H. Rizwan M. Fahad S.. (2019). Seed priming with melatonin coping drought stress in rapeseed by regulating reactive oxygen species detoxification: antioxidant defense system, osmotic adjustment, stomatal traits and chloroplast ultrastructure perseveration. Ind. Crops Prod. 140, 111597. doi: 10.1016/j.indcrop.2019.111597

[B30] Kim W. Y. Ali Z. Park H. J. Park S. J. Cha J. Y. Perez-Hormaeche J.. (2013). Release of SOS2 kinase from sequestration with GIGANTEA determines salt tolerance in *Arabidopsis* . Nat. Commun. 4, 1352. doi: 10.1038/ncomms2357 23322040

[B31] Kołodziejczyk I. Bałabusta M. Szewczyk R. Posmyk M. M. (2015). The levels of melatonin and its metabolites in conditioned corn (*Zea mays* L.) and cucumber (*Cucumis sativus* L.) seeds during storage. Acta Physiol. Plant. 37, 105. doi: 10.1007/s11738-015-1850-7

[B32] Li W. Wang Z. Cao J. Dong Y. Chen Y. (2023). Melatonin improves the homeostasis of mice gut microbiota rhythm caused by sleep restriction. Microbes Infect. 25, 105121. doi: 10.1016/j.micinf.2023.105121 36804006

[B33] Li D. Wei J. Peng Z. Ma W. Yang Q. Song Z.. (2020). Daily rhythms of phytomelatonin signaling modulate diurnal stomatal closure via regulating reactive oxygen species dynamics in *Arabidopsis* . J. Pineal. Res. 68, e12640. doi: 10.1111/jpi.12640 32064655

[B34] Liang D. Ni Z. Xia H. Xie Y. Lv X. Wang J.. (2019). Exogenous melatonin promotes biomass accumulation and photosynthesis of kiwifruit seedlings under drought stress. Sci. Hortic. 246, 34–43. doi: 10.1016/j.scienta.2018.10.058

[B35] Lichtenthaler H. K. Wellburn A. R. (1983). Determinations of total carotenoids and chlorophylls a and b of leaf extracts in different solvents. Biochem. Soc Trans. 11, 591–592. doi: 10.1042/bst0110591

[B36] Liu Y. Li H. Xiao Y. Xu X. Lin L. Liao M.. (2022). Exogenous melatonin treatments promote the growth and nutrient uptake of peach (*Prunus davidiana*) seedlings. J. Plant Nutr. 45, 2292–2302. doi: 10.1080/01904167.2022.2063737

[B37] Lv Y. Pan J. Wang H. Reiter R. J. Li X. Mou Z.. (2021). Melatonin inhibits seed germination by crosstalk with abscisic acid, gibberellin, and auxin in *Arabidopsis.* Journal of pineal research. J. Pineal. Res. 70, e12736. doi: 10.1111/jpi.12736 33811388

[B38] Ma Z. Liu X. Liu Y. (2022). Studies on the biosynthetic pathways of melanin in *Auricularia auricula* . J. Basic Microbiol. 62, 843–856. doi: 10.1002/jobm.202100670 35419841

[B39] Matschi S. Vasquez M. F. Bourgault R. Steinbach P. Chamness J. Kaczmar N.. (2020). Structure-function analysis of the maize bulliform cell cuticle and its potential role in dehydration and leaf rolling. Plant Direct. 4, e00282. doi: 10.1002/pld3.282 33163853 PMC7598327

[B40] Meftahizadeh H. Baath G. S. Saini R. K. Falakian F. Hatami H. (2023). Melatonin-mediated alleviation of soil salinity stress by modulation of redox reactions and phytochemical status in guar (*Cyamopsis tetragonoloba* L.). J. Plant Growth Regul. 42, 4851–4869. doi: 10.1007/s00344-022-10740-z

[B41] Mir A. R. Siddiqui H. Alam P. Hayat S. (2020). Melatonin modulates photosynthesis, redox status, and elemental composition to promote growth of *Brassica juncea*-a dose-dependent effect. Protoplasma 257, 1685–1700. doi: 10.1007/s00709-020-01537-6 32778964

[B42] Moderie C. Boudreau P. Shechter A. Lespérance P. Boivin D. B. (2021). Effects of exogenous melatonin on sleep and circadian rhythms in women with premenstrual dysphoric disorder. Sleep 44, 171. doi: 10.1093/sleep/zsab171 PMC8664575 34240212

[B43] Nakano Y. Asada K. (1981). Hydrogen peroxide is scavenged by ascorbate specific peroxidase in spinach chloroplasts. Plant Cell Physiol. 22, 867–880. doi: 10.1093/oxfordjournals.pcp.a076232

[B44] Pathan A. K. Bond J. Gaskin R. E. (2010). Sample preparation for SEM of plant surfaces. Mater. Today 12, 32–43. doi: 10.1016/S1369-7021(10)70143-7

[B45] Reiter R. J. (1991). Pineal melatonin: cell biology of its synthesis and of its physiological interactions. Endocr. Rev. 12, 151–180. doi: 10.1210/edrv-12-2-151 1649044

[B46] Robinson A. R. Mansfield S. D. (2009). Rapid analysis of poplar lignin monomer composition by a streamlined thioacidolysis procedure and near infrared reflectance based prediction modeling. Plant J. 58, 706–714. doi: 10.1111/j.1365-313X.2009.03808.x 19175772

[B47] Song Z. Yang Q. Dong B. Li N. Wang M. Du T.. (2022). Melatonin enhances stress tolerance in pigeon pea by promoting flavonoid enrichment, particularly luteolin in response to salt stress. J. Exp. Bot. 73, 5992–6008. doi: 10.1093/jxb/erac276 35727860

[B48] Steed G. Ramirez D. C. Hannah M. A. Webb A. A. R. (2021). Chronoculture, harnessing the circadian clock to improve crop yield and sustainability. Science 372, eabc9141. doi: 10.1126/science.abc9141 33926926

[B49] Su C. Wang P. Wu J. Wang H. Fan J. Gong W.. (2023). Effects of melatonin on the photosynthetic characteristics of *zanthoxylum armatum* under waterlogging stress. Russ. J. Plant Physiol. 70, 82. doi: 10.1134/S1021443722602129

[B50] Thangaraj K. Liu S. Li J. Zhao Z. Han R. Mei H.. (2022). Exogenous melatonin alleviates sooty mould on tea plants (*Camellia sinensis* L.). Sci. Hortic. 299, 111056. doi: 10.1016/j.scienta.2022.111056

[B51] Usanmaz S. Akn M. A. Kahramanoglu (2022). Effects of foliar melatonin application on the flowering and fruit set of pomegranate (*Punica granatum* L. ‘Wonderful’). Acta Hortic. 1349), 63–71. doi: 10.17660/ActaHortic.2022.1349.10

[B52] Wang Q. An B. Wei Y. Reiter R. J. Shi H. Luo H.. (2016). Melatonin regulates root meristem by repressing auxin synthesis and polar auxin transport in *Arabidopsis* . Front. Plant Sci. 7. doi: 10.3389/fpls.2016.01882 PMC5156734 28018411

[B53] Wang X. He Y. Wei H. Wang L. (2021). A clock regulatory module is required for salt tolerance and control of heading date in rice. Plant Cell Environ. 44, 3283–3301. doi: 10.1111/pce.14167 34402093

[B54] Wang Y. Zhao H. Hu X. Zhang Y. Zhang Z. Zhang L.. (2022). Transcriptome and hormone analyses reveal that melatonin promotes adventitious rooting in shaded cucumber hypocotyls. Front. Plant Sci. 13. doi: 10.3389/fpls.2022.1059482 PMC9742233 36518515

[B55] Wei H. Kong D. Yang J. Wang H. (2020). Light regulation of stomatal development and patterning: shifting the paradigm from *Arabidopsis* to grasses. Plant Commun. 1, 100030. doi: 10.1016/j.xplc.2020.100030 33367232 PMC7747992

[B56] Wei W. Tao J. Yin C. Chen S. Zhang J. Zhang W. (2022). Melatonin regulates gene expressions through activating auxin synthesis and signalling pathways. Front. Plant Sci. 13. doi: 10.3389/fpls.2022.1057993 PMC9792792 36582645

[B57] Wei L. Zhao H. Wang B. Wu X. Lan R. Huang X.. (2021). Exogenous melatonin improves the growth of rice seedlings by regulating redox balance and ion homeostasis under salt stress. J. Plant Growth Regul. 41, 2108–2121. doi: 10.1007/s00344-021-10417-z

[B58] Xu Y. Zhang J. Wan Z. Huang S. Di H. He Y.. (2023). Physiological and transcriptome analyses provide new insights into the mechanism mediating the enhanced tolerance of melatonin-treated rhododendron plants to heat stress. J. Integr. Agric. 22, 2397–2411. doi: 10.1016/j.jia.2023.07.005

[B59] Yang N. Han M. Teng R. Yang Y. Wang Y. Xiong A.. (2022). Exogenous melatonin enhances photosynthetic capacity and related gene expression in a dose-dependent manner in the tea plant (*Camellia sinensis* (L.) Kuntze). Int. J. Mol. Sci. 23, 6694. doi: 10.3390/ijms23126694 35743137 PMC9223723

[B60] Yang X. Ren J. Li J. Lin X. Xia X. Yan W.. (2023). Meta-analysis of the effect of melatonin application on abiotic stress tolerance in plants. Plant Biotechnol. Rep. 17, 39–52. doi: 10.1007/s11816-022-00770-0

[B61] Yang L. Sun Q. Wang Y. Chan Z. (2021). Global transcriptomic network of melatonin regulated root growth in *Arabidopsis* . Gene. 764, 145082–145092. doi: 10.1016/j.gene.2020.145082 32858176

[B62] Zahedi S. M. Hosseini M. S. Hoveizeh. N. F. Gholami R. Abdelrahman M. Tran L. P. (2021). Exogenous melatonin mitigates salinity-induced damage in olive seedlings by modulating ion homeostasis, antioxidant defense, and phytohormone balance. Physiol. Plant. 173, 1682–1694. doi: 10.1111/ppl.13589 34716914

[B63] Zhang M. Gao C. Xu L. Niu H. Liu Q. Huang Y.. (2022c). Melatonin and indole-3-acetic acid synergistically regulate plant growth and stress resistance. Cells 11, 3250. doi: 10.3390/cells11203250 36291118 PMC9600385

[B64] Zhang T. Wang Y. Ma X. Ouyang Z. Deng L. Shen S.. (2022b). Melatonin alleviates copper toxicity via improving ROS metabolism and antioxidant defense response in tomato seedlings. Antioxidants 11, 758. doi: 10.3390/antiox11040758 35453443 PMC9025625

[B65] Zhang G. Yan Y. Zeng X. Wang Y. Zhang. Y. (2022a). Quantitative proteomics analysis reveals proteins associated with high melatonin content in barley seeds under NaCl-induced salt stress. J. Agric. Food Chem. 70, 8492–8510. doi: 10.1021/acs.jafc.2c00466 35759742

[B66] Zia S. F. Berkowitz O. Bedon F. Whelan J. Franks A. E. Plummer K. M. (2019). Direct comparison of Arabidopsis gene expression reveals different responses to melatonin versus auxin. BMC Plant Biol. 19, 567. doi: 10.1186/s12870-019-2158-3 31856719 PMC6921455

