# Supplementary material for: Overexpression of oHIOMT results in various morphological, anatomical, physiological and molecular changes in switchgrass
Source: Front Plant Sci. 2024 Jun 17;15:1379756. doi: 10.3389/fpls.2024.1379756 (PMC11215127; doi:10.3389/fpls.2024.1379756)
Supplement: Supplementary file 1 [file Table_1.docx]

Supplementary Table 1 Primer sequences used in the experiments

| **Gene name** | **Forward primer (5'-3')** | **Forward primer (5'-3')** |
| --- | --- | --- |
| *β-Actin* | TTCGCTCTTCCTCACGC | GATGTCACGGACGATTTC |
| *CER1* | CATACGGCACTTCATTCGCTGTTG | CGCTTCCTTCTCCCAAGACAATCC |
| *FAR2* | CAGCGTATGTGAATGGGCAGAGG | ACTGATCCTTGGCTATGGTGTCTCC |
| *KCS6* | AACGTCGGGATCAACCTGAACAAG | CGATGAAGGAGAGCGCGAACAG |
| *KCS19* | GGACGAGCACTACCCCAACTCC | GAGGTGGTAGCACTGCTTGAGC |
| *FDH1* | GTGTTCTACAAGGCTGGCGAGTAC | AATGTAGTGATGGCCCTGTGATTCG |
| *MnSOD* | CTGGAAGAACCTCAAGCCTATTAGCG | ACAAGTGCCTCAAATGAACCAAAATCC |
| *FeSOD* | AGCCAATTGCCAGCCTAGACTTATG | GTGACAGTGTCCCAAGAGATGAGATG |
| *CAT* | ACAGGCAGGAGAGGTTCGTCAG | CGTCGCACTTTGAGAGCAGGTC |
| *APX* | GCCGCCTTCCTGATGCTACTAAG | GATCGCTCCTTGTGGCACCTTC |
| *SOS1* | AGGGAGAGGGAGATGGGTGAG | GACGCCGAGGACGAGAAGG |
| *NHX1* | CGTGGTGGCGCACCTGGT | TGCAGAAAAGATCGCTCC |
| *HKT4* | TCAGCATTTCTGCCAACAAC | GGCCAAGATAGTGCGAGAAC |
| *HAK5* | AATCGTACCAAGAGGGCTCA | ACGCCATCACCAATTACCAT |
| *HAK7* | GCGGAGTACTTCTTGCCATC | TGCAGCACAAGACAGGGATA |
| *HAK27* | TCTCCCTTGGTGGCATACTC | TGATGGGACTAAACCGAAGC |
| *GI* | GGAGAGTCAACCAGCGAATAGTG | ACCGTCAACAAGCATTCCATCAG |
