# Supplementary material for: Overexpression of oHIOMT results in various morphological, anatomical, physiological and molecular changes in switchgrass
Source: Front Plant Sci. 2024 Jun 17;15:1379756. doi: 10.3389/fpls.2024.1379756 (PMC11215127; doi:10.3389/fpls.2024.1379756)
Supplement: Supplementary file 2 [file Table_2.docx]

Supplementary Table 2 Anatomical structure analysis of transgenic switchgrass

| **Observation traits/abbreviation** | **WT** | **MMT** | **MRT** |
| --- | --- | --- | --- |
| Area of abaxial epidermis cells (ABE, μm2) | 134.4±5.9 **b** | 243.7±24.8 **a** | 108.2±15.0 **b** |
| Area of adaxial epidermal cells (ADE, μm2) | 156.4±12.7 **b** | 239.9±18.9 **a** | 102.9±8.4 **c** |
| Area of bundle sheath cells (BSCs, μm2) | 501.1±23.9 **b** | 1093.1±153.7 **a** | 423.7±32.4 **b** |
| Area of bulliform cells (BCs, μm2) | 778.4±68.2 **b** | 1109.2±135.5 **a** | 489.4±53.6 **c** |
| Diameter of vascular bundles (VBs, μm) | 153.5±9.3 **b** | 201.0±8.3 **a** | 115.3±13.8 **c** |
| Area of xylem vessels (XVs, μm2) | 766.2±48.7 **b** | 1073.1±118.2 **a** | 573.6±46.1 **c** |
| Number of BSCs in single bundle | 11±1.3 **a** | 12±1.6 **a** | 13±1.0 **a** |
| Number of VBs of per cross-section | 81±7.9 **a** | 87±4.6 **a** | 93±2.6**a** |

Note: WT: wild type; MMT: melatonin-moderate transgenic switchgrass; MRT: melatonin-rich transgenic switchgrass. Values represent means ± SE; Different letters within each row indicate a significant difference at *p* < 0.01.
