# Supplementary material for: Overexpression of oHIOMT results in various morphological, anatomical, physiological and molecular changes in switchgrass
Source: Front Plant Sci. 2024 Jun 17;15:1379756. doi: 10.3389/fpls.2024.1379756 (PMC11215127; doi:10.3389/fpls.2024.1379756)
Supplement: Supplementary file 3 [file DataSheet_1.pdf]

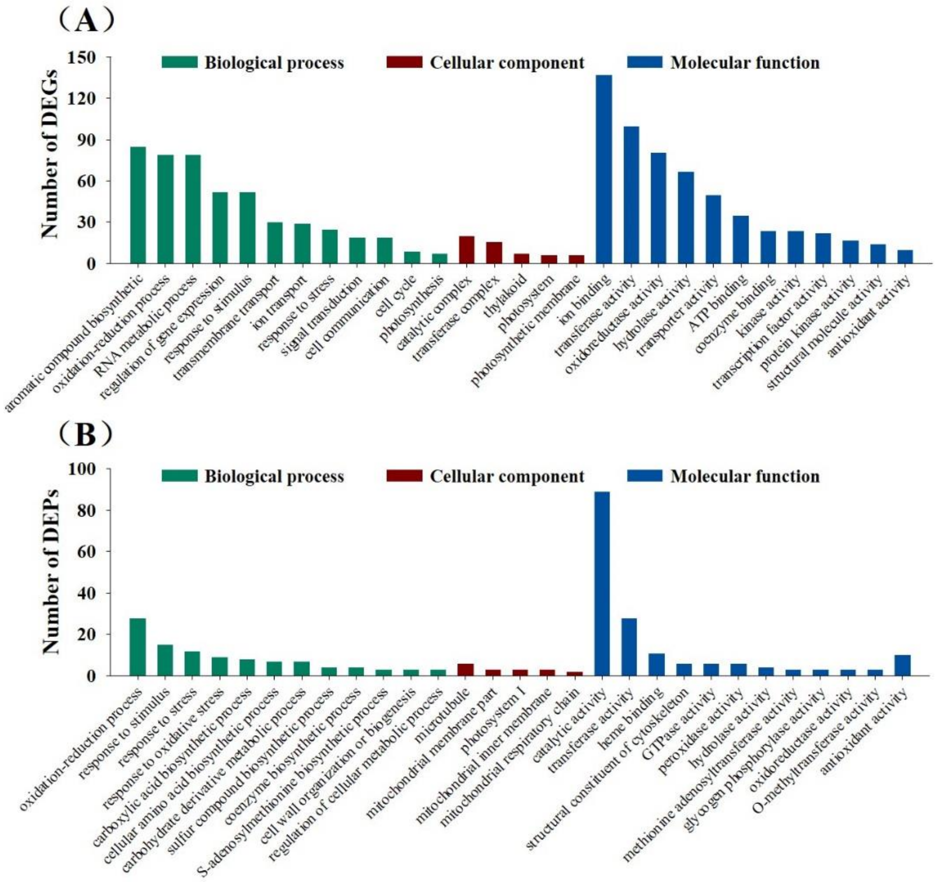

Supplementary Figure 1. GO classifications of DEGs (A) and DEPs (B) between MMT and MRT plants. MMT: melatonin-moderate transgenic switchgrass; MRT: melatonin-rich transgenic switchgrass; DEG: differentially expressed gene; DEP: differentially expressed protein.
