# Supplementary material for: Overexpression of oHIOMT results in various morphological, anatomical, physiological and molecular changes in switchgrass
Source: Front Plant Sci. 2024 Jun 17;15:1379756. doi: 10.3389/fpls.2024.1379756 (PMC11215127; doi:10.3389/fpls.2024.1379756)
Supplement: Supplementary file 4 [file DataSheet_2.pdf]

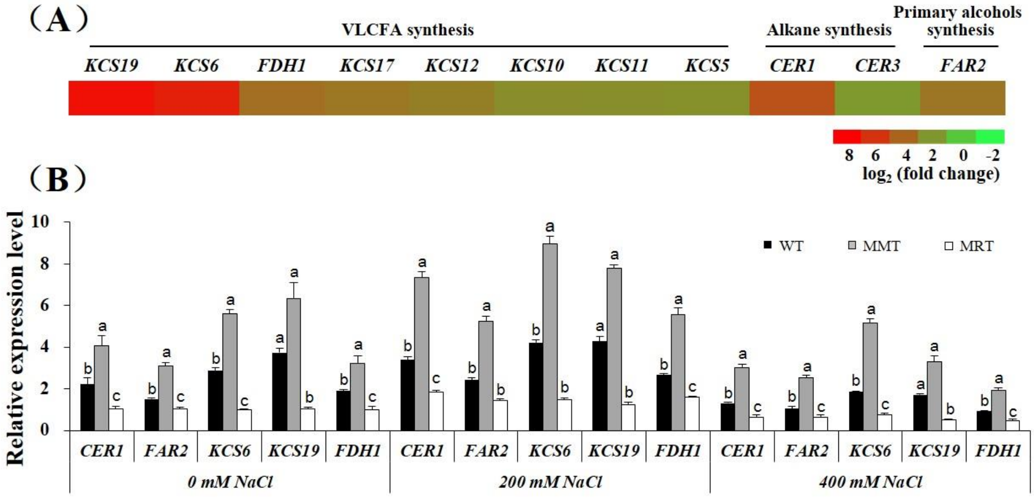

Supplementary Figure 2. Expression profiles of genes related to wax biosynthesis in MMT plants compared with MRT plants under normal (A) and salt stress conditions (B). WT: wild type; MMT: melatonin-moderate transgenic switchgrass; MRT: melatonin-rich transgenic switchgrass; Values are means ±SE (n=3); Different letters above the bars indicate significant differences at $p < 0.01$ (Duncan's test).
