# Supplementary material for: Overexpression of oHIOMT results in various morphological, anatomical, physiological and molecular changes in switchgrass
Source: Front Plant Sci. 2024 Jun 17;15:1379756. doi: 10.3389/fpls.2024.1379756 (PMC11215127; doi:10.3389/fpls.2024.1379756)
Supplement: Supplementary file 5 [file DataSheet_3.pdf]

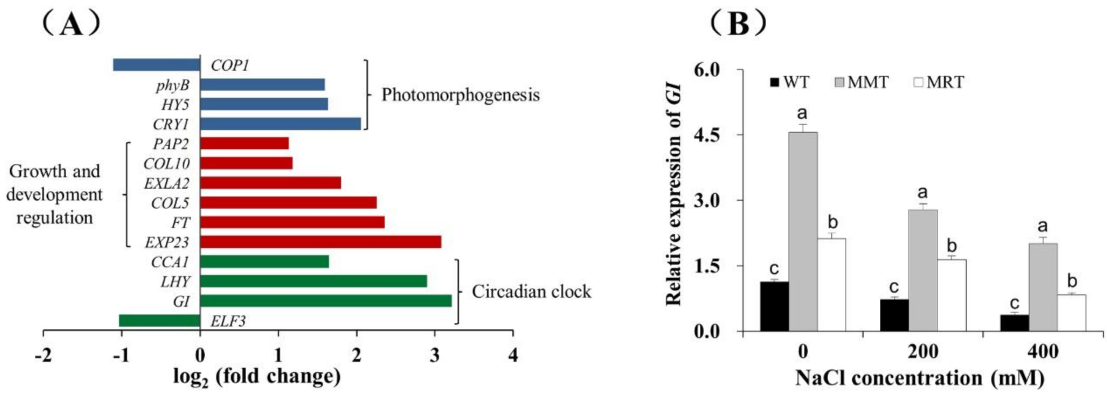

Supplementary Figure 3. Effects of melatonin on circadian rhythm. (A) DEGs involved in circadian rhythm-plant pathway in MMT plants compared with MRT plants. (B) Expression levels analysis of circadian clock output gene GI under salt stress. WT: wild type; MMT: melatonin-moderate transgenic switchgrass; MRT: melatonin-rich transgenic switchgrass; DEG: differentially expressed gene; Values are means ± SE (n=3); Different letters above the bars indicate significant differences at $p < 0.01$ (Duncan's test).
